# Ground condition as a risk factor in sports injury aetiology studies: the level of concordance between objective and subjective measures

**DOI:** 10.1186/s40621-014-0027-y

**Published:** 2014-12-15

**Authors:** Dara M Twomey, Lauren A Petrass, John W Orchard, Caroline F Finch

**Affiliations:** 1 Faculty of Health, Federation University Australia, Mt Helen Campus, Ballarat, 3353 VIC Australia; 2 Australian Centre for Research into Injury in Sport and its Prevention (ACRISP), Federation University Australia, Ballarat, 3353 VIC Australia; 3 School of Public Health, University of Sydney, Sydney, 2222 NSW Australia

**Keywords:** Ground hardness, Injury aetiology, Injury prevention, Injury risk factors, Subjective ground rating, Objective ground measures

## Abstract

**Background:**

It is well known that the condition and type of sporting ground influences the risk of sports injury. However, the lack of evidence on the relationship between subjective and objective sporting ground condition assessments in sports injury aetiology studies has implications for the development of effective injury prevention strategies. This paper aims to examine concordance between subjectively rated and objective ground hardness and moisture measurements to inform data collection methods for future sports injury aetiology studies.

**Methods:**

Subjective, observational assessments of ground hardness and soil moisture were recorded on 36 occasions during an Australian football season using two four-point scales of ‘very soft’ to ‘very hard’ and ‘very wet’ to ‘very dry’, respectively. Independent, objectively measured hardness and soil moisture were also undertaken at nine locations on the same grounds. The maximum and minimum ground values and the computed average of ground hardness and soil moisture were analysed. Somer’s d statistic was calculated to measure the level of concordance between the subjective and objective measures.

**Results:**

A significant, moderate to substantial level of agreement was found between the subjective ratings and the average objective hardness values (*d* = 0.467, *p* <0.001), but there was perfect agreement on just less than half of the occasions. The level of concordance between the subjective and objective moisture ratings was low to moderate or trivial for all moisture measures (0.002 < *d* <0.264, *p* >0.05).

**Conclusions:**

Compared to objective measures, the subjective assessments were more accurate for ground hardness than for soil moisture levels and raters were just as likely to underestimate or overestimate the condition under review. This has implications for future sports injury aetiology studies that include ground condition assessments and particularly the use of subjective measures to underpin the development of future injury prevention strategies.

**Electronic supplementary material:**

The online version of this article (doi:10.1186/s40621-014-0027-y) contains supplementary material, which is available to authorized users.

## Background

Understanding the mechanisms and factors contributing to sport injury risk is critical in the development of effective injury prevention strategies (Bahr, Krosshaug [2005]). The association between sports injury risk and ground conditions has been reported, particularly in the different types of football (Lee, Garraway [2000]; Twomey et al. [2012a]; Hagel et al. [2003]). Ground hardness (representing the scale from very soft to very hard) has been the most cited ground condition related to sports injury (Orchard [2002]), but despite the quantity of studies, the veritable link between ground hardness and injury risk has not been well established. The dearth of high quality evidence, coupled with inconsistencies in the descriptors used to subjectively measure ground hardness, has largely been responsible for this (Petrass and Twomey [2013]). Subjective measures have most commonly been collected through self-report, survey questions or by visual observation of sporting grounds. A limitation of the subjective ground studies is that they could be subject to bias and confounding due to factors which have not been measured and reported in previous studies. These factors include such things as the footwear worn by the assessor, the specific locations assessed on the ground, or innate perceptual differences between observers (Petrass and Twomey [2013]). More objective measures conducted with devices specifically developed to measure surface properties, such as the Clegg Impact Soil Tester (SD Instrumentation Ltd., Bath, UK), are considered to provide more accurate assessments of ground conditions (Twomey et al. [2011]) but have rarely been included in sports injury aetiology studies. Knowledge of whether subjective measures accurately reflect objective measures of ground hardness is needed. This knowledge would then inform appropriate ground condition data collection in sports injury surveillance studies that aim to determine the relationship between ground hardness and sports injury risk.

To date, 27 studies have associated sports injury rates or risk with ground hardness, primarily in different types of football including American football, (Ramirez et al. [2006]; Adkison et al. [1974]) Australian football, (McMahon et al. [1993]; Twomey et al. [2012a]) soccer (Chomiak et al. [2000]; Ekstrand, Gillquist [1983]) and rugby union/league (Takemura et al. [2007]; Gabbett et al. [2007]; Dallalana et al. [2007]). The majority (22 studies) based their conclusions about the relationship with injury risk on subjective ratings (e.g. coach reports) of ground hardness, and their findings remain inconclusive despite some evidence to suggest that harder/drier grounds compared to softer/wetter grounds result in an increase in minor injuries (Petrass, Twomey [2013]). The major limitation of these studies using subjectively based measures as a key risk factor is the inconsistent terms used to describe ground hardness. For example, in addition to using the description ‘hard’, ill-defined terms such as ‘wet’ and ‘dry’ or their derivatives have also been frequently used to classify ground hardness (Adkison et al. [1974]; Bramwell et al. [1972]), and in some instances, a combination of terms have been used, i.e. ‘wet/soft’ and ‘dry/hard’ (Cromwell et al. [2000]). While a relationship between objectively measured soil moisture and ground hardness has been reported (Baker [1991]), subjectively rated moisture levels have never been compared to objectively rated measures. Overall, the lack of detail regarding how the subjective assessments were conducted also makes it difficult to compare across studies, and the lack of consistency in results raises the question of the reliability and validity of the subjective assessments and hence, the quality of the reported links to injury risk (Petrass and Twomey [2013]).

Another issue with studies that have subjectively rated grounds is the lack of information on variability across the ground and whether the assessment gives an accurate representation of the entire ground (Petrass and Twomey [2013]). The condition of natural grass playing surfaces can be influenced by such factors as where the players undertake their training and how much they do, the level of play (e.g. elite sport versus community sport) and maintenance of the grounds; these can vary across a ground. Therefore, reporting overall terms such as ‘hard’ or ‘heavy’ may not accurately reflect the condition of all parts of the ground. Consequently, linking injury risk to an overall subjective rating during training or games, without matching the injury to the ground condition at the exact site of the injury, may be erroneous (Petrass and Twomey [2013]).

In contrast to the extensive literature on injury risk and subjectively rated ground hardness, only five sports injury risk studies have objectively obtained ground hardness/softness measures (Takemura et al. [2007]; Twomey et al. [2012b]; Twomey et al. [2012a]; Orchard et al. [2005]; Norton et al. [2001]). Overall, these studies did not find a strong association between hard grounds and an increased injury risk. However, two of the studies postulated that harder grounds may indirectly contribute to injury risk through enabling players to run faster resulting in higher impact forces should they collide during play (Norton et al. [2001]; Takemura et al. [2007]). A study in a senior community-level Australian football found that despite the low number of injuries on very hard or low-normal grounds, the relative risk of injury was significantly higher in both of these categories compared to the preferred hardness range (Twomey et al. [2012a]). In contrast to findings using subjective assessments (Alsop et al. [2005]; Gabbett et al. [2007]; Dallalana et al. [2007]), a non-significant relationship between ground hardness and injury in rugby union was found when using objective ground measures (Takemura et al. [2007]). With such inconsistencies in results, comparisons of subjective and objective measures in the same study would provide valuable information on the accuracy and validity of subjective measures.

Objectively measuring ground conditions is more expensive and time-consuming than merely conducting observations and is not possible in all studies. However, unlike subjective ratings, the reliability of objective ground measures has been established (Twomey et al. [2011]). Until the level of agreement between subjectively and objectively rated ground hardness has been determined, it is difficult to have confidence in the relationships between injury risk and ground hardness being reported from subjective assessments. Consequently, the development of injury prevention strategies based on subjective assessments of ground hardness may not be appropriate or effective. Therefore, the aim of this paper is to examine the level of concordance between subjectively rated ground hardness and moisture levels and objective ground measurements on the same natural grass surfaces and to make recommendations for the types of measures to include in future sports injury epidemiology studies.

## Methods

This study was nested within the Preventing Australian Football Injuries through Exercise (PAFIX) group-clustered randomised controlled trial (cRCT) and involved both subjective and objective assessments of ground hardness and soil moisture in a sample of the sporting grounds from Victoria (Australia) where the trial was conducted. Full details of the PAFIX project have been published elsewhere (Finch et al. [2009]) and institutional ethical approval was granted by the University of Ballarat Human Ethics Committee. Details of how the grounds were sampled for this ground assessment sub-study have also been published (Twomey et al. [2012a]).

The subjective/observational measurements were recorded prior to every game by trained primary data collectors (PDCs). All PDCs received formal training on how to undertake the ground assessment (i.e. recommended pathway for the assessment), how to record the data and were instructed to complete the assessment approximately 20 min before the game started to avoid collisions with the players warming-up. The PDCs evaluated and recorded the hardness of the ground according to a four-point scale of ‘very soft’, ‘soft’, ‘hard’, and ‘very hard’ and the ground’s moisture level on both grassed and bare areas according to ‘very wet’, ‘wet’, ‘dry’, and ‘very dry’.

Objective hardness and soil moisture measurements were collected at nine locations (Twomey et al. [2012a]) on the same grounds the day before matches by an independent experienced operator. The hardness readings were taken from a single drop of a 2.25 kg Clegg hammer, released from 45 cm through a guide tube and deceleration on impact in gravities (g) was recorded. The reliability of the ground hardness measures and full details of the assessment protocol have been previously reported (Twomey et al. [2011]; Twomey et al. [2012a]). Soil moisture content was measured using a HydroSense Moisture Meter (Campbell Scientific Inc., Logan, UT, USA) with two 12 cm probe rods. The percentage of volumetric water content was recorded at a shallow level (45 degree angle) and a deep level (90 degree angle) at each of the nine locations on the ground. The measurements at the nine locations were averaged to give an overall objective ground average for the two properties. The maximum and minimum hardness and moisture values across the nine locations for each ground assessment were also identified and used in the analyses to assess if difference in agreement existed with the extremes compared to the average values.

Overall, it was possible to directly pair 36 subjective and objective assessments of sporting grounds. There were no changes in weather conditions that would have influenced the ground conditions between the objective and subjective assessments for these 36 pairs. Due to the continuous nature of the objective data and the categorical nature of the subjective data, frequency distributions were conducted and matching categories were established for the objective data. Cross tabulations were calculated, and a Somer’s d statistic was computed to measure the level of concordance between the subjective and objective measures for both hardness and soil moisture. The Somer’s d was used as it is a measure of association for a contingency table when the rows and columns represent ordered categories (Everitt [1995]). To establish the strength of these relationships, published correlation coefficient ranges were used: *r* = 0.01 to 0.09, trivial; *r* = 0.10 to 0.29, low to moderate; *r* = 0.30 to 0.49 moderate to substantial; *r* = 0.50 to 0.69, substantial to very strong; *r* = 0.70 to 0.89, very strong; and *r* = 0.90 to 0.99, near perfect (de Vaus [2002]).

## Results

**Figure 1:**
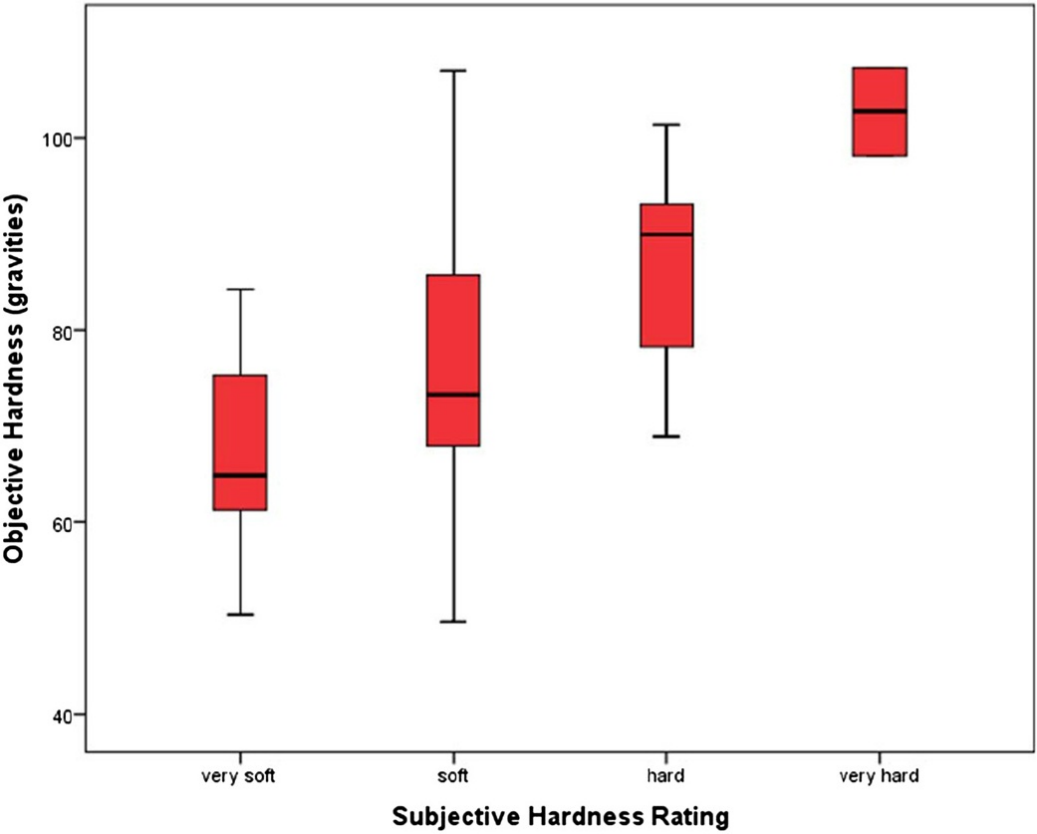
**Box-and-whisker plot representing the hardness values for the four categories of subjective rating.** The median is depicted by the solid horizontal line in the box and the maximum and minimum values by the whiskers.

**Figure 2:**
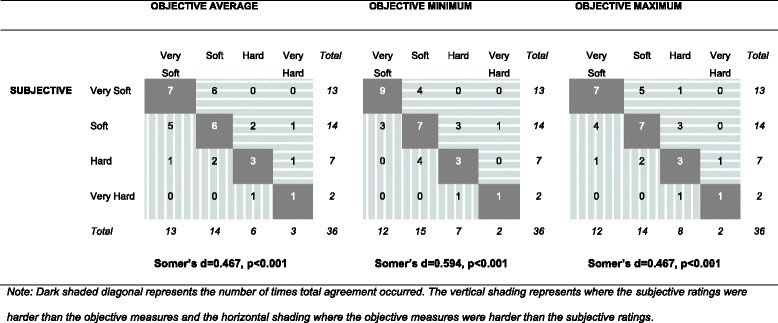
**Level of agreement between the subjective hardness ratings and the objective hardness measures (** ***n*** **= 36 assessment pairs).**

As higher objective hardness values represent harder grounds, the increase in the median value from very soft through to very hard in Figure 1 demonstrates that the subjective ratings were able to distinguish soft and hard grounds. The greater variation, evident by the length of the whiskers in the box and whisker plot, shows that grounds at the extreme ends of soft or hard were more accurately rated. The level of agreement between the subjective rating of ground hardness and the objectively measured hardness is presented in Figure 2.

Dark shaded diagonal areas represent the number of times total agreement occurred. The vertical shading represents where the subjective ratings were harder than the objective measures, and the horizontal shading represents where the objective measures were harder than the subjective ratings.

Overall, the assessments of subjective ratings and the average objective values agreed 47% of the time (*n* = 17), and the level of concordance was moderate to substantial (*d* = 0.467, *p* <0.001). The subjective assessments rated the ground as less hard than the average objective measure in ten (28%) instances and as harder in nine (25%) cases. The highest level of agreement was found when the ground was ‘very soft’ (53.8%, *n* = 7/13).

When the ratings were collapsed into two levels, soft/very soft and hard/very hard, on both types of assessments, the agreement between the subjective and average objective assessments increased to 83% (*n* = 30/36). The disagreements, however, were equally likely to be due to subjective assessments that over- or underestimated ground hardness, compared to the objective assessments (*n* = 3, each).

When comparing the subjective ratings to the maximum objective hardness value, the percentage of agreement was 50% (*n* = 18) and the level of concordance remained as moderate to substantial. However, the subjective ratings and the minimum objective assessment value agreed 55% of the time (*n* = 20), and the level of concordance was substantial to strong (*d* = 0.594, *p* <0.001). Similar to the average objective measures, the highest level of agreement was for the ‘very soft’ category for both minimum (*n* = 9/13) and maximum (*n* = 7/13) hardness.

**Figure 3:**
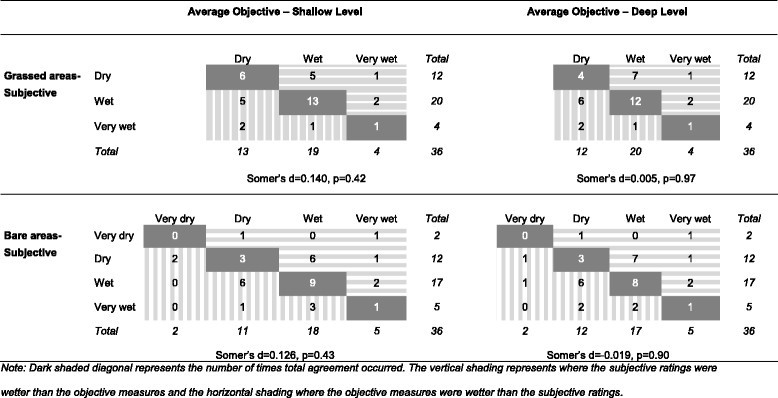
**Level of agreement between the grassed and bare subjective moisture ratings and the shallow and deep average objective hardness measures (** ***n*** **= 36 pairs).**

The level of agreement between the subjective and objective average moisture ratings are presented in Figure 3. There were no occasions where the grassed areas were subjectively rated as ‘very dry’, reducing the classifications to three categories. The level of agreement was higher for both assessments of grassed areas (56% for shallow level, 47% for deep level) than of bare areas (36% for shallow level, 33% for deep level). In most comparisons, the subjective assessment was just as likely to under- or overreport the presence of wet conditions. As evident from Figure 3, the level of concordance between the subjective and objective moisture ratings was low to moderate or trivial for both shallow and deep moistures.

Dark shaded diagonal areas represent the number of times total agreement occurred. The vertical shading represents where the subjective ratings were wetter than the objective measures, and the horizontal shading represents where the objective measures were wetter than the subjective ratings.

## Discussion

Extremes in climatic conditions, particularly over the past decade, have resulted in an increasing amount of literature being published on the association between ground conditions and injury risk and the need to reduce/prevent such injuries (Ramirez et al. [2006]; Takemura et al. [2007]; Twomey et al. [2012a]). Subjectively rated ground hardness has been linked to injury risk in many sports injury epidemiology studies; however, the strength of the potential injury risk factors is dependent on the quality and accuracy of the measures used, and the validity of subjective assessment in this context is yet to be reported (Petrass and Twomey [2013]). This is the first study to compare subjective and objective ground assessments, and the findings suggest that the ability to accurately rate ground hardness and moisture level subjectively may be difficult. Overall, the results show a greater level of agreement between objective and subjectively rated ground hardness compared to soil moisture levels. However, for both ground conditions, when the objective and subjective assessments did not agree on scales needing a high level of differentiation (e.g. soft vs. very soft), they were just as likely to under- or overestimate the ground condition under review. This finding has implications for future recommendations and the use of subjective assessment measures over more accurate but costly objective ground measures in sports injury epidemiology studies.

Ground hardness refers to the ability of the surface to absorb impact forces (Orchard et al. [1999]) and has been reported in previous sports injury studies using a variety of subjective rating scales. The most common has been a simple dichotomous scale of ‘hard’ or ‘soft’ (Ryan, McQullian [1992]; Inglis, Stewart [1979]) to more complex scales including aspects of traction as well as hardness, for example, ‘hard’, ‘firm’, ‘yielding’, ‘slippery’ or ‘heavy’ (Lee, Garraway [2000]). In this study, the four-point scale of ‘very hard’, ‘hard’, ‘soft’ or ‘very soft’ was selected for reasons of simplicity and its use in previous work (Alsop et al. [2005]). The results show that the subjective assessments of a ground rated as soft correlated well with the combined average objective ratings as either ‘soft’ or ‘very soft’ and a hard ground as ‘hard’ or ‘very hard’. While this is a pleasing result, perfect agreement was only reached in approximately half of the 36 assessments when categories were not combined. Given the inability of the subjective raters to distinguish between ‘soft’ and ‘very soft’ and ‘hard’ and ‘very hard’, replacing ‘very’ with ‘unacceptably’ might be valuable in future assessments. To ensure there is accuracy in the association between subjectively rated ground conditions and sports injury, it is critical that the reliability and validity of any subjective scale are established and reported in future studies or, at the very least, the limitations of the subjective scale acknowledged. Ideally, the standardisation of ground condition measurement scales/categories would be valuable for comparisons between findings of different studies. It is also essential that the descriptors represent the particular ground condition under review, for example, hardness or traction, to ensure that injury risk is accurately associated with the specific ground property.

Even though a subjective ground assessment is generally based on the average condition of the whole ground, this study also investigated whether the level of agreement was stronger with extremes of objective measures such as the maximum or minimum hardness values of each ground. A slightly higher level of agreement was found between the subjective ratings and the minimum hardness objective values than with the average or maximum values. This result was based on the increased agreement of 9/13 for the ‘very soft’ ratings with minimum hardness objective values compared to 7/13 for the same category with the average or maximum objective values. Regardless of which objective measure was used, the findings show that the subjective and objective ratings only perfectly matched on approximately 50% to 60% of the occasions. It is questionable if this is a sufficient level of agreement to have confidence in reports linking subjective ratings of ground hardness to injury risk and indicates that, where possible, objective measures or proven valid and reliable subjective measures should be used in future sports injury epidemiology studies.

In the agronomic literature, the level of soil moisture has been linked with ground hardness (Baker [1991]), and it has also been used in subjective ratings of ground conditions in sports injury epidemiology studies (Hagel et al. [2003]). It has even been suggested that soil moisture could be used as a proxy measure for ground hardness, given the expensive and time-consuming nature of objectively measuring ground hardness. The findings of this study show a very low level of concordance between subjectively rated and objectively measured soil moisture levels and therefore adds doubt to some of the previous studies purporting the link between sports injury risk and soil moisture (Cromwell et al. [2000]; Hagel et al. [2003]; Andresen et al. [1989]). It was anticipated that it would be easier to accurately assess the moisture level on the more worn, bare ground areas. However, there was slightly higher agreement for the grassed areas than the bare areas. These soil moisture findings have implications for the use of subjective soil moisture ratings in future injury-related research, and it is recommended that hardness rather than soil moisture ratings be used in such studies.

The strength of this study is that it was conducted over a full playing season which resulted in a range of sporting ground conditions. It is acknowledged, however, that differences in rainfall and more extreme conditions could be experienced in other playing seasons or other regions. As this study was embedded in the PAFIX project, there were multiple subjective raters involved. The results presented do not discern between the raters and so it is possible that some raters were more accurate at assessing grounds than others. Having said that, the data collection process was deliberately chosen to mirror what happens in the normal sports injury studies context where, before a given game, different raters would assess and record the condition of the ground they are assigned to determine if a given playing surface was safe for play.

A possible limitation of this study is that it was not feasible to independently observe each subjective assessment to ensure the instructions were being followed correctly. Hence, it relied on the integrity of the PDCs to undertake a thorough walk around the ground prior to making the assessment each time. The instructions and training provided to the PDCs included going through each term in the subjective assessment and providing the recommended pathway to walk around the ground. In this study, no psychometric testing of the data collectors was undertaken. The inclusion of such tests to assess the suitability of data collectors may lead to increased reliability and validity of subjective assessment of ground conditions in future studies. Training the data collectors on grounds of varying conditions might also prove valuable in future work. While the use of objective measures is the most ideal option, where it is not possible, some standardisation of subjective category labels in future sports injury epidemiology studies will help to improve the accuracy of results provided by subjective ground assessments and lead to greater confidence in using the injury risk results to inform injury prevention strategies.

## Conclusions

Overall, when compared to objective measures, the subjective assessments were more accurate for ground hardness than soil moisture levels but raters were just as likely to under- or overestimate the condition under review. The low relationship between subjective and objective assessments for moisture is alarming given the reliance on this type of ground condition assessment in studies that consider injury risk factors. If subjective ground assessments are to be used in future epidemiological studies which aim to establish an association with injury risk, there is a need to standardise practices and to ensure that the reliability of assessors and details of the assessed locations are stated.

## Authors’ original submitted files for images

Below are the links to the authors’ original submitted files for images. Authors’ original file for figure 1 Authors’ original file for figure 2 Authors’ original file for figure 3

## Abbreviations

PAFIX: Preventing Australian Football Injuries through Exercise
cRCT: group-clustered randomised controlled trial
PDC: Primary data collectors
