# Supplementary figures and images for: Ground condition as a risk factor in sports injury aetiology studies: the level of concordance between objective and subjective measures
Source: Inj Epidemiol. 2014 Dec 15;1(1):27. doi: 10.1186/s40621-014-0027-y (PMC5005679; doi:10.1186/s40621-014-0027-y)

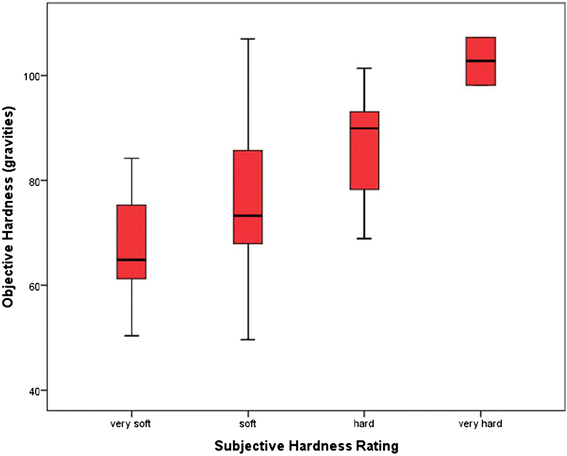

Supplement: Supplementary file 1 — Authors’ original file for figure 1 [file 40621_2014_27_MOESM1_ESM.gif]

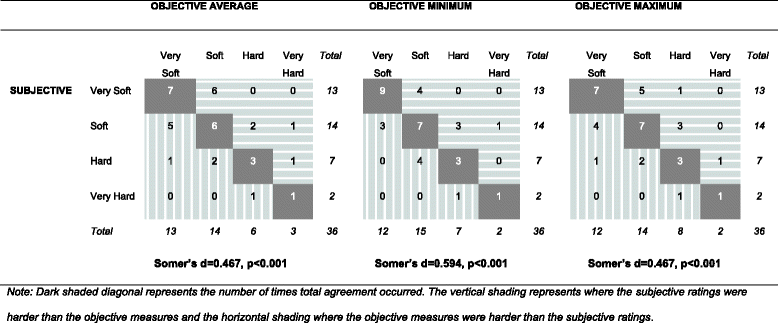

Supplement: Supplementary file 2 — Authors’ original file for figure 2 [file 40621_2014_27_MOESM2_ESM.gif]

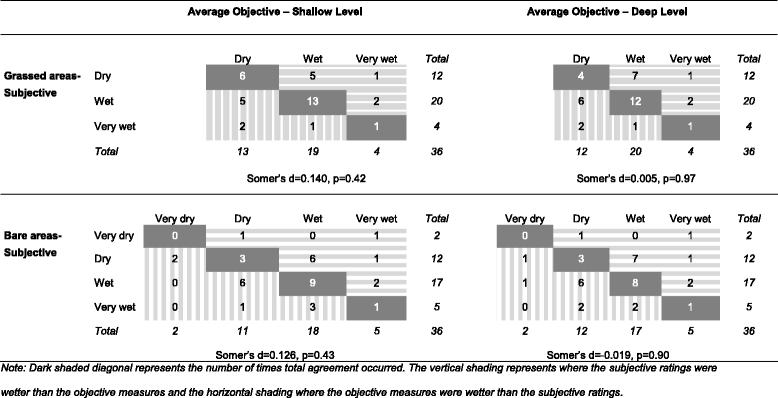

Supplement: Supplementary file 3 — Authors’ original file for figure 3 [file 40621_2014_27_MOESM3_ESM.gif]
